# Gain of 17q in malignant fibrous histiocytoma is associated with a longer disease-free survival and a low risk of developing distant metastasis

**DOI:** 10.1038/sj.bjc.6601069

**Published:** 2003-08-12

**Authors:** W-H Weng, J Åhlén, W-O Lui, O Brosjö, S-T Pang, A von Rosen, G Auer, O Larsson, C Larsson

**Affiliations:** 1 Department of Molecular Medicine, Karolinska Hospital CMM L8:01, SE-171 76 Stockholm, Sweden; 2 Department of Surgery, Karolinska Hospital P9:03, SE-171 76 Stockholm, Sweden; 3 Department of Orthopedics, Karolinska Hospital, SE-171 76 Stockholm, Sweden; 4 Department of Oncology and Pathology, CCK R8:04, Karolinska Hospital, SE-171 76 Stockholm, Sweden

**Keywords:** sarcoma, comparative genomic hybridisation, malignant fibrous histiocytoma, prognosis, chromosome 17

## Abstract

In this study, a panel of 39 primary malignant fibrous histiocytomas (MFH) of high malignancy grade were characterised for chromosomal alterations. The results were then evaluated in relation to the survival and the occurrence of recurrent disease during follow-up for an average period of 63 months. Chromosomal alterations detected by comparative genomic hybridisation (CGH) were recorded in 37 of the 39 cases analysed. The most frequent CGH abnormalities were gains of 17p, 20q, 16p, 17q, 1p31, 7q21, and 9cen-q22, and losses of 9p21-pter and 13q21–22. However, the patterns of CGH imbalances did not allow the identification of a single common event, suggesting that the key initiating event(s) is not a numerical imbalance. Patients with tumours harbouring a gain of 17q showed significantly longer overall and disease-free survival (*P*=0.001 and 0.008) as well as lower frequency of metastasis (*P*=0.018) during follow-up. Taken together, the findings suggest that the clinical outcome of MFH is associated with the genetic profiles of the primary tumours. Importantly, a subgroup of MFHs characterised by a low risk of developing metastasis and local recurrence is recognised based on their frequent gains of 17q by CGH.

Malignant fibrous histiocytoma (MFH) is a type of soft tissue sarcoma (Enzinger and Weiss, 2001), typically showing a clinically aggressive behaviour with frequent development of distant metastases and local recurrences after surgery. Microscopically, MFHs are recognised as pleomorphic tumours composed of fibroblasts, myofibroblasts, and histiocytes (Enzinger and Weiss, 1995); however, the cellular origin is still unclear. So far, the parameters size, localisation, necrosis, intravascular invasion, and histopathological characteristics of the tumour have been demonstrated to have prognostic value (Gustafson, 1994; Scully *et al*, 1994; Singer *et al*, 1994; Tanabe *et al*, 1994; Coindre *et al*, 1996; Le Doussal *et al*, 1996; Fletcher *et al*, 2001; Gibbs *et al*, 2001). However, these parameters are not specific and sensitive enough to allow the identification of a patient group at high risk of developing metastases and local recurrences. Therefore, the development of additional and objective prognostic markers would be of obvious clinical value in treatment planning.

The MFH entity is today regarded as a heterogenous group of tumours, and from its initial introduction more than 20 years ago, the relative frequency of soft tissue sarcomas diagnosed as MFH has varied, both between clinical units and during time (Fletcher *et al*, 2001). Hence, further elucidation of the genetic profiles of tumours diagnosed as MFH are expected to contribute to the improved understanding of aetiological and prognostic aspects of this tumour group. Cytogenetic data on MFHs are so far limited, and the reported karyotypes are often complex with multiple numerical and structural abnormalities. No specific recurrent chromosomal translocation has been identified so far in MFH, but recurrent breakpoints in 1p36, 1q11, 1q21, 3p12, 11p11, 17p11, and 19p13 have been reported (Rydholm *et al*, 1990; Choong *et al*, 1996; Mertens *et al*, 1998). Additional chromosomal aberrations have been identified in studies using comparative genomic hybridisation (CGH) (Larramendy *et al*, 1997; Mairal *et al*, 1999; Parente *et al*, 1999). The recurrent gains of chromosomal regions 1p31 and 7q32 were significantly associated with a poor overall survival and poor metastasis-free or overall survival, respectively (Larramendy *et al*, 1997). In agreement with the CGH findings of 12q gain, candidates within this chromosomal region, such as sarcoma amplification sequence (SAS), human homologue of murine double minutes 2 (MDM2), cyclin-dependent kinase 4 (CDK4), and high-mobility group protein IC (HMGIC) have been shown to be amplified in MFH by molecular analyses (Nilbert *et al*, 1995). More recently, an amplicon derived from 8p23.1 was found to be amplified and overexpressed in MFHs, and from this area a candidate gene designated MASL1 was subsequently isolated (Sakabe *et al*, 1999). Frequently lost regions including 9p21 and 13q14-q21 pinpointed the candidates p16^INK4A^ (9p21) and RB1 (13q14), which were subsequently shown to be deleted by molecular approaches (Simons *et al*, 2000).

Here, a series of primary MFH cases of high malignancy grade were characterised genetically, and the findings were then evaluated in relation to the clinical outcome regarding survival and development of recurrent disease during follow-up.

## MATERIALS AND METHODS

### Patients and tumour samples

The study includes 39 MFH cases identified from all patients treated for a primary soft tissue sarcoma of high malignancy grade at the Karolinska Hospital between 1986 and 1993 (Åhlén *et al*, in preparation). All patients were retrospectively followed up until October 2001 or until the patient's death (mean 63; range 3–134 months), and the occurrence of metastasis and/or local recurrence was recorded. None of the patients had received preoperative or postoperative chemotherapy or radiation treatments, none was diagnosed with local or distant metastases at the time of the initial surgery, and none died from intercurrent disease within 3 years after the diagnosis. The diagnosis of MFH was established at histopathological re-evaluation of paraffin sections stained with haematoxylin and eosin by one of the authors. The classification followed the published criteria (Enzinger and Weiss, 1995), and was based on the exclusion of other types of sarcomas in combination with the recognition of a heterogeneous morphology typical of MFH with characteristic myxoid, pleomorphic or storiform growth pattern or the presence of giant cells. When necessary, the differential diagnostics to other types of soft tissue sarcomas were supported by the results from immunostainings according to the published recommendations (Weiss, 1994; Enzinger and Weiss, 1995). All cases were classified as being of high-malignancy grade, that is, grade 3 or 4 on a four-graded scale and which is based on the estimation of cellularity, cellular atypia, necrosis, and mitotic frequency (Angervall *et al*, 1986).

### DNA extractions

DNA was extracted from the 39 frozen tumour samples obtained at surgery and stored at −70°C. At the time of the initial collection, a representative imprint was performed from each case and stained with Feulgen. Re-evaluation of these slides confirmed that the tumours contained a clear majority of tumour cells (>70%), and were therefore suitable for CGH analysis. From each of these frozen samples, five 8 *μ*m consecutive sections were cut and used for DNA extraction using the QIA amp DNA Mini Kit (QIAGEN, Hiden, Germany) according to the procedure suggested by the manufacturer. For 10 of the cases, DNA was also extracted from sections of the paraffin blocks used for the histopathological re-evaluation.

### Comparative genomic hybridisation

Comparative genomic hybridisation was performed on DNA extracted from the 39 frozen MFH tumours, and for 20 of the tumours the experiments were performed in duplicate with identical results. As an external control for the tumour samples, DNAs from corresponding paraffin blocks were analysed in parallel for 10 of the cases, which always gave the same results. In control experiments, normal male DNA was hybridised against normal female DNA, and as a negative control DNA from histopathologically normal tissue adjacent to a tumour was hybridised. Comparative genomic hybridisation was performed essentially as described (Kallioniemi *et al*, 1994). In short, genomic tumour DNAs were directly labelled by nick-translation using fluorescence isothiocyanate 12-dUTP (DuPont NEN, Boston, MA, USA). The nick-translation conditions were adjusted to obtain DNA fragments ranging from 300 to 2000 bp. Spectrum Red labelled normal reference DNA (Vysis Inc., Downers Grove, IL, USA) was used as control DNA. The tumour DNA, normal reference DNA, and unlabelled Cot-1 DNA (Life Technologies, Gaithersburg, MD, USA) were mixed with 10 *μ*l of hybridisation solution (50% formamide, 10% dextran sulphate in 2 × SSC), denatured, and applied onto denatured normal metaphase slides (Vysis Inc.). Hybridisation was performed at 37°C for 48–72 h, following which the slides were washed, dried, and counterstained with 0.1 *μ*g/ml 4,6-diamidino-2-phenylindole (DAPI, Sigma Inc.) in an anti-fade solution (Vectashield, Vector Inc.).

### Digital image analyses

In all, 10 three-colour digital images (DAPI, FITC, and Spectrum Red fluorescence) were captured from each experiment using a Zeiss Axioplan 2 imaging (Carl Zeiss Jena GmbH, Jena, Germany) epifluorescence microscope and analysed with the isis/CGH software (Metasystems). For each tumour DNA hybridisation, at least 12 ratio profiles were averaged for each chromosome to reduce noise. The chromosomal regions were interpreted as lost when the green-to-red ratio was less than 0.75, and as gained if the ratio was greater than 1.25. A high-level amplification was defined if the ratio exceeded 2.0. The analyses of the profiles observed in 1p and 16p were interpreted with caution, in particular, for the low level of imbalances. Heterochromatic regions, the short arm of the acrocentric chromosomes, chromosome Y, and the GC-rich chromosome regions 19 and 22q known to give some false-positive results, were excluded from the evaluation.

### Statistical analyses

Correlations between the total number of CGH alterations, as well as the most common alterations of individual chromosomes were evaluated in relation to the outcome during follow-up using the Kaplan–Meier survival test and the log rank test. The comparisons were made against patients who were without evidence of disease at the end of follow-up. To evaluate if the significant CGH changes were independent of the already known prognostic parameters of tumour size, tumour depth, and high mitotic index, necroses multivariate analyses with Cox regression analyses were used. Owing to the limited number of cases analysed, the different variables were compared in pairs in the multivariate analyses. The calculations were performed in Statistica 6.0 Software, and probabilities of less than 0.05 were accepted as significant.

## RESULTS

In this study, a series of 39 primary MFH tumors (Table 1

**Table 1:** Summary of clinical data of the 39 cases of MFH in the study

		**Recurrent disease**		**End of follow-up**			**5-year survival**	
	**Informative**	**Rec/Met**	**DF**	**DOD**	**NED**	**DFS mean**	**DFS**	**OAS**
**Variable**	**(*n*=)**	**(*n*=)**	**(*n*=)**	**(*n*=)**	**(*n*=)**	**(months)**	**(%)**	**(%)**
*Sex*								
Female	20	13	7	11	9	36	37	55
Male	19	10	9	8	11	45	45	55
*Malignancy grade*								
Grade III	14	10	4	6	8	35	34	59
Grade IV	25	13	12	13	12	40	44	50
*Depth*								
Intramuscular	15	10	5	9	6	32	32	43
Subcutaneous	9	2	7	0	9	—	66	70
Extracompartmental	12	8	4	7	5	33	23	43
*Size (cm)*								
>8	19	15	4	13	6	24	18	36
⩽8	19	7	12	5	14	—	66	72
*Surgical margin*								
Wide	18	10	8	7	11	46	43	58
Marginal	10	4	6	3	7	—	57	65
Intralesional	7	5	2	5	2	26	14	29
*Site*								
Upper extremity	3	2	1	1	2	—	17	19
Lower extremity	24	14	10	12	12	36	38	58
Trunk	7	4	3	4	3	16	23	32
Pelvis	5	3	2	2	3	—	19	24
*Age at diagnosis (years)*								
>60	26	13	13	11	15	69	52	60
⩽60	13	10	3	8	5	16	23	43
								
Total	39	23	16	19	20	40	43	56

DF=disease free; DOD=dead of disease; NED=no evidence of disease; DFS=disease-free survival; OAS=overall survival.

) were screened for genomic imbalances using CGH. The clinical data for the cases studied are summarised in Table 1. Of the 39 cases, 20 were female and 19 were male, and the median age at the initial surgery was 69 years (range 22–82). All patients had been surgically treated and the tumours were removed with the best possible margin. The patients were retrospectively followed up, during which recurrent disease in the form of local recurrence and/or distant metastases were recorded in 23 patients. At the end of follow-up, 19 patients had died from the disease, while 20 were without evidence of the disease.

The CGH alterations detected in the individual tumours are detailed in Table 2

**Table 2:** The most frequent CGH alterations in relation to the total number of alterations detected in the 39 MFH

	**No. of**	**Gain involving chromosomal regions**										
**Case no**	**changes**	**20q**	**17p**	**7q21**	**1p31**	**1q23–24**	**17q**	**9cen-q22**	**16p**	**Other gains**	**Other losses**	
1	0											
2	0											
3	1	20q										
4	1		17p									
5	1		**17p**									
6	1									12p		
7	1									4p		
8	1			7q								
9	1										4q	
10	1										1q41-qter	
11	1										20p	
12	1									12q14–15		
13	1				1p31–q24	1p31–q24						
14	2	20q								10p		
15	2		17p				17q			Xp		
16	2							9cen-q22		Xq,		
17	2			7cen-q31					16p			
18	2									1p32–34	9p13-pter	
19	2	20q					17q					
20	3		17p					9q		15q24-qter		
21	3										11, 14q, 16q	
22	4	20q	17p							12q12–15	9p	
23	4	20q							16p	7p	X	
24	5									11cen-q13	4, 5q14–q23, 13q14–32 , 6q14–q16	
25	5	20q	17p		1p31-pter	1q11–24	17q			5p,12q23-qter, 18p		
26	5		17p		1p31-pter		17q		16p	9q34-qter, 11cen-q13		
27	6	20q					17q	9q	16p	6p	13q	
28	7		17p					9q		8q21.1–q22, 12q11–15,	8p21-pter, 20p	
29	7		17p	7cen-q21			17q		16p	2p22-pter, 6p, 7p, 9q33-qter	13q12–31	
30	7		17p			**1q23–32**	17q		16	6p24–26, 6q24-26, 12q13–21	9p	
31	8		17p	7q11.1-21					16p	10p, 11cen-q13, 13q14-qter, Xpter-q21	8q22-qter	
32	8		17p		1p31-pter		17q	9q	16p	6q22–24, 13q31-qter, 18p	8,	
33	9	20q	17p	7q			17q	9q		6p21.3-pter, 6q24-qter, 7p, 15q, 18p	3q	
34	9	20q					17q			4p15.3-pter, 5p, 10p, 11cen-q13	3q, 6q11–21, 9p21-pter	
35	11	20q	17p	7q	1p31-pter		17q		16p	7p, 11p14-q13, 18p	4, 6q, 10, 13q14-qter	
36	11	20q		**7q21–32**	1cen-p31			9cen-q22	16p	4q13–24, 11cen-q13	1q21-qter, 9p, 13q, 11q22-qter	
37	12	20q	17p	7cen-q21	1p31-pter	1q23–25		9q	16p	7p, 8p11.2–12, 11cen-q14, Xp, 13q22-qter, 19q12–13.2		
38	15	20q	17p		1p21-pter	1cen-q32	17q		16p	2q12.2–21, 6p21.1–21.3, 6q24-qter8p, 15q, 18, Xp	4q21-qter, 6q12–22, 11q21-qter	
39	16	20q	17p	7cen-q32	1p	1cen-q31		9q		2p13–q21, 3q, 5p, 6, 8q24.1-qter,12q, 21q, **Xp**	2q33-qter, 3p23-pter, 10q23-qter	

Bold letter represents high-level amplification.

**Figure 1:**
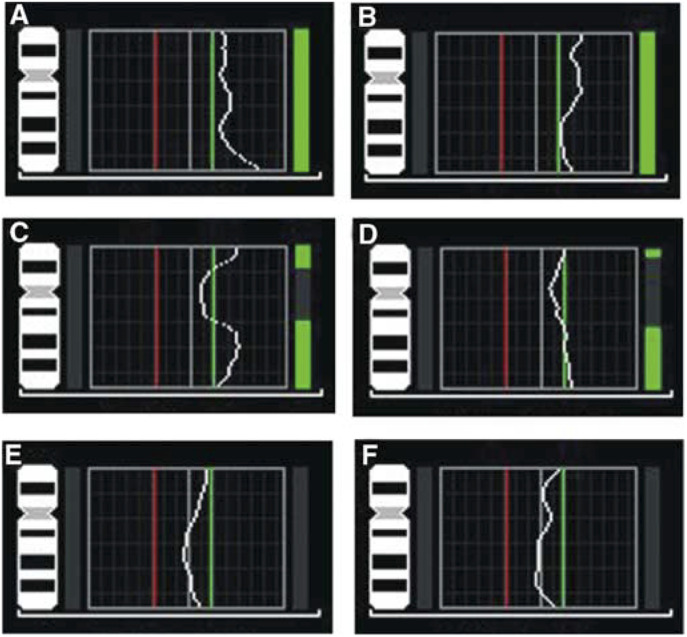
CGH profiles demonstrating tumours with (**A–D**) and without (**E** and **F**) copy number gains including chromosome 17q in MFH tumours.

**Figure 2:**
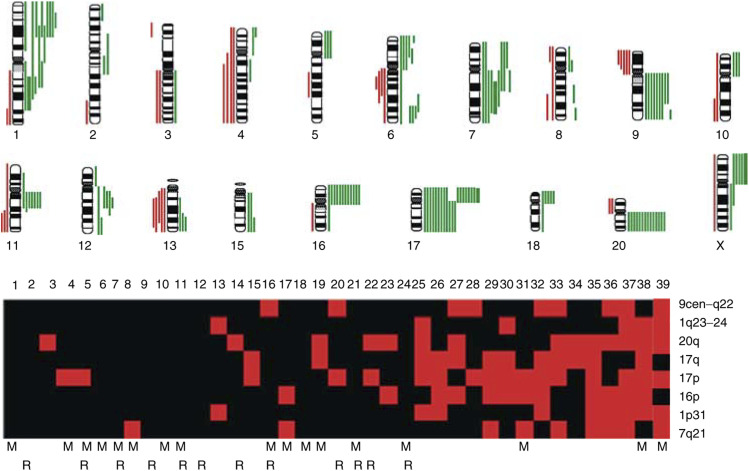
A summary of DNA sequence number alterations detected by CGH in at least two of the 39 MFH tumours studied as shown at the top. One alteration identified in one tumour is represented by one line, with losses indicated to the left and gains to the right of the ideograms. High-level amplifications of subchromosomal regions are marked with thick lines. The distribution of the CGH alterations detected in MFH tumours for the most frequently involved subchromosomal regions are illustrated at the bottom. Red boxes indicate a gain in one tumour, while black boxes represent tumours without a CGH imbalance in the particular region. The cases are ordered 1–39 from left to right, and the patients who developed distant metastases and/or local recurrences during follow-up are marked by M and R, respectively.

. An example of the obtained CGH profiles is shown in Figure 1

, and the subchromosomal regions involved are illustrated in Figure 2

. Overall, genetic imbalances were found in all chromosomes analysed, and gains were more frequently detected than losses. Gains were commonly found at chromosomal regions 17p (17 out of 39), 20q (14 out of 39), 16p and 17q (12 out of 39), 1p31, 7q21, and 9cen-q22 (9 out of 39) (Figure 2, Table 2). In addition, high copy number gains were observed in three tumours, each involving a different subchromosomal region, that is 1q23–32, 7q21–32, and Xp (Figure 2, Table 2). Losses were most frequently detected in the subchromosomal regions 9p21-pter and 13q21–22 (five out of 39) in this series (Figure 2, Table 2). Some alterations were found to occur as single alterations, while others were only recognized in tumours with several other changes. However, the patterns of CGH imbalances did not allow the identification of a single common event.

**Figure 3:**
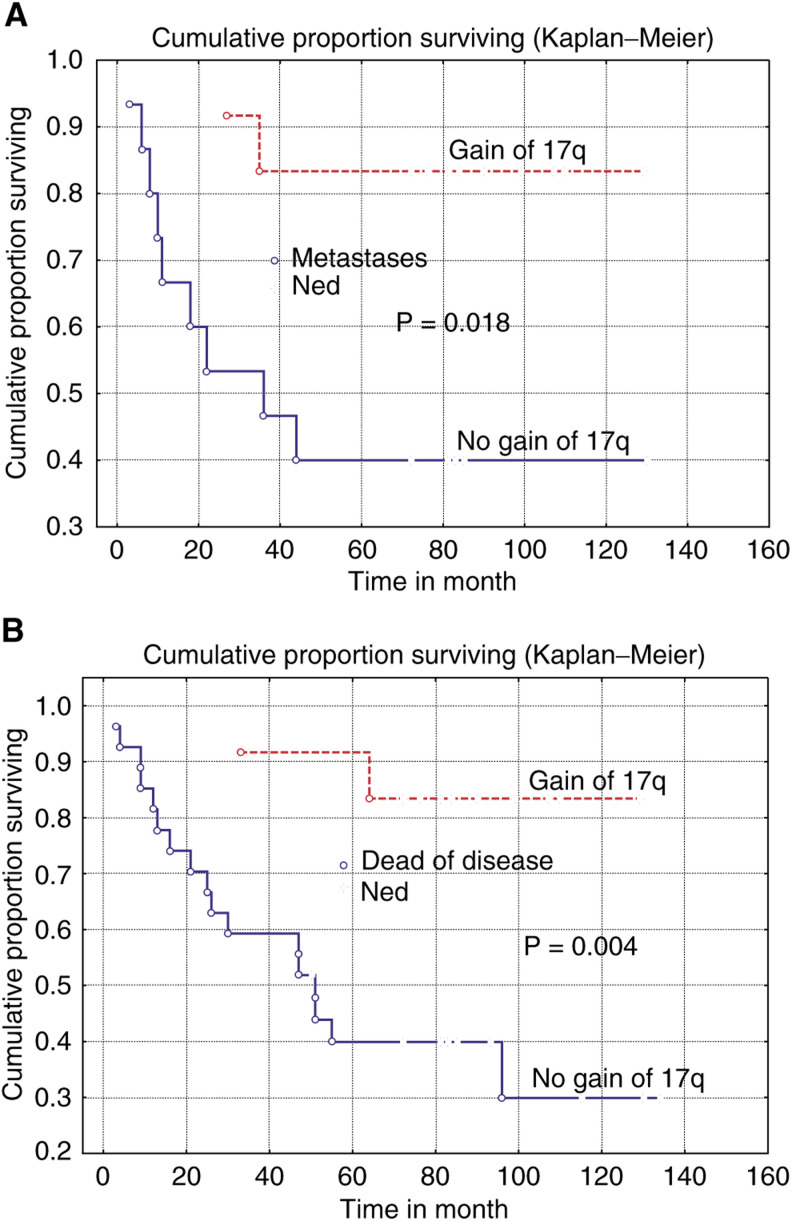
Kaplan–Meier curves demonstrating the significant associations between gain of 17q and longer disease-free survival (**A**), and a lower frequency of metastasis without local recurrence (**B**), during follow-up of patients with highly malignant MFH tumours. The comparisons were made against cases who were without evidence of the disease at the end of follow-up.

Of the 39 tumors analysed, 37 displayed at least one CGH alteration and the number of detected changes varied from 1 to 16 in the individual tumours. When compared to the sizes of the tumours, no association to the total number of CGH alterations was observed. The total numbers of CGH alterations as well as the most commonly involved subregions (16p, 17q, 1p31, 7q21, 9cen-q22, 17p, and 20q) were then evaluated in relation to the survival and the development of metastases only or in combination with local recurrence during follow-up. From these comparisons, several interesting associations were revealed. We found that cases with four or more alterations were significantly associated with longer overall (*P*=0.07) and disease-free survival (*P*=0.018), and with lower frequency of metastases only (*P*=0.009) or in combination with local recurrence (*P*=0.036) during follow-up. Most significantly, gain of 17q was strongly associated with longer overall survival (*P*=0.001) and disease-free survival (*P*=0.004, Figure 3

), as well as with a lower frequency of metastases only (*P*=0.018, Figure 3) or in combination with local recurrence (*P*=0.01) during follow-up. Following the identification of 17q gain as significantly associated with prognosis in univariate analyses, multivariate analyses was used to demonstrate that gain of 17q is independent of other known prognostic factors including tumour size, tumour site, and mitotic index (*P*-values between 0.01 and 0.04).

## DISCUSSION

Gross chromosomal alterations leading to numerical imbalances were shown to be present in the vast majority of MFH. Some alterations were found to occur as a single alteration, while others were only recognised in tumours with several other changes. However, neither in the tumours with few nor in tumours with multiple changes did the individual CGH profiles allow the identification of a single common event. This implies that alterations beyond the detection level of CGH are important for early tumorigenesis, such as translocations, inversions or other balanced alterations. As illustrated in Figure 2, several chromosomal regions tended to be altered together in the same tumour, possibly reflecting a functional relationship between the alterations or a genetic heterogeneity of the MFH entity or both.

MFH tumours are generally of high malignancy grade (i.e. 3 or 4 on a four-graded scale), and the survival rate varies from 50 to 70% (Rooser *et al*, 1991; Pritchard *et al*, 1993; Le Doussal *et al*, 1996). However, for patients with metastatic or recurrent disease, the expected survival is very poor (Enzinger and Weiss, 2001). Therefore, the recognition of patients with tumours diagnosed as highly malignant MFH, and which are at risk of developing metastasis and local recurrence, is essential for their proper clinical handling and treatment. At best, this distinction of a high-risk group should be performed by the analyses of markers on tumour specimens from the primary surgery, and using a robust and reproducible methodology. The observation that the different types of CGH profiles for primary MFH tumours were associated with the clinical outcome of the MFH disease during follow-up represents a first step towards these goals. Specifically, we found that patients with gain of 17q in their tumours had significantly improved disease-free survival, and rarely developed metastases during follow-up (Figure 3). Although the findings are statistically significant, they were based on a limited number of cases, and should therefore be confirmed in a larger sample series.

In this study, we have chosen to evaluate the CGH imbalances in relation to the survival and the development of metastases only or in combination with local recurrence. The development of local recurrence only would be another highly relevant parameter of the clinical outcome. However, since the extent of the surgical margin should optimally be taken into account in the evaluations, a larger series of MFH cases would be required to allow separate analyses of patients with wide, marginal, and intralesional excision. Given these uncertainties, it can still be noted that gain of 17q and 16p were both associated with a lower occurrence of local recurrence only during follow-up (*P*=0.011 and 0.030, respectively). Similarly, cases with gain of 16p, 17p, and 20 developed significantly lower frequencies of metastases in combination with local recurrences (*P*=0.02, 0.048, and 0.020, respectively).

For several types of sarcomas, the detailed elucidation of genetic changes in primary tumours have identified alterations associated with the outcome during follow-up (Ladanyi *et al*, 2002; Mertens *et al*, 2002). The findings in this study demonstrate that the clinical outcome for patients with highly malignant MFH is associated with the genetic profiles of the primary tumours. On a purely speculative basis, we could consider that one MFH subgroup would be genetically characterised by gain of 17q, and clinically by a less aggressive course with a low risk of metastases and local recurrences, and hence a better survival. The second subgroup would be clinically characterised by an aggressive course with frequent development of distant metastases and local recurrences. The lack of recurrent genetic abnormality by CGH in this latter group would then imply a structural abnormality in the aetiology. In further support of this hypothesis, recurrent structural alterations involving chromosomes 1, 3, 11, 17, and 19 have been reported in MFH, some of which have been associated with a high risk of recurrence and metastases (Rydholm *et al*, 1990; Choong *et al*, 1996; Mertens *et al*, 1998). Finally, an alternative, but less likely, explanation would be that the high- and low-risk groups could have common unidentified initiating genetic events, and that the varying clinical course results from secondary chromosomal imbalances such as gain of 17q in the low-risk group. Regardless of the mechanism involved, detailed elucidation of the structural abnormalities in cultured MFH tumours would be worthwhile to proceed using a molecular cytogenetic approach. Furthermore, it is envisaged that the exact identification of the putative oncogene activation driving the 17q gain will be valuable for genetic dissection of the MFH entity, and in addition will enable the development of additional prognostic markers for clinical practice.
